# Unanticipated gastroduodenal fistula induced by multiple magnetic beads: successful endoscopic management without the need for surgery

**DOI:** 10.1055/a-2578-2330

**Published:** 2025-04-17

**Authors:** Xing Wang, Haifeng Liu, Zhujun Gu, Kai Lin, Weiwei Cheng, Ling Wang, Haijun Zhang

**Affiliations:** 1 Department of Digestive Endoscopy Center, Shanghai Childrenʼs Hospital, Shanghai Jiaotong University School of Medicine, Shanghai, China

A 3-year-old boy was referred to our hospital following the incidental discovery of foreign bodies on a chest computed tomography scan, initially performed to investigate episodes of intermittent coughing. The exact timing of the ingestion remains indeterminate. Abdominal examination identified mild tenderness in the upper abdomen without signs of peritoneal irritation. Abdominal X-ray showed four foreign bodies in the upper abdomen, with no signs of pneumoperitoneum ( Fig. 1 ). Initially, we believed that the four connected foreign bodies were retained in the stomach, as their total length reached nearly 5.5 cm. However, findings from the gastroscopy were unexpected.

**Fig. 1:**
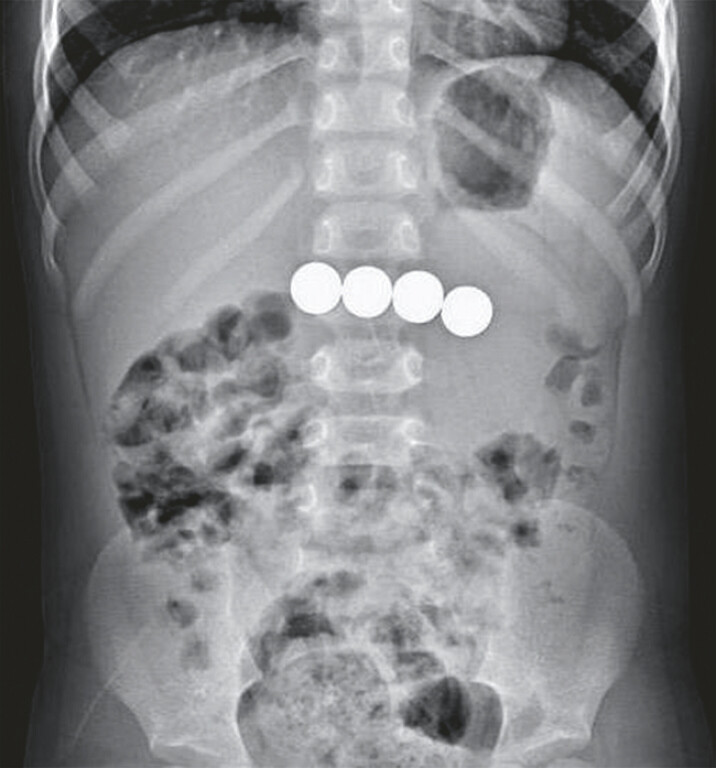
An abdominal X-ray reveals the presence of four spherical foreign bodies retained in the upper abdomen, with no evidence of pneumoperitoneum.

Gastroscopy revealed two magnetic beads located on the lesser curvature of the stomach and another two located in the duodenal bulb, tightly attracted to each other. Endoscopic removal was then performed meticulously; the first magnetic bead in the stomach was smoothly retrieved using an endoscopic retrieval net, while the second bead was largely embedded in the gastric mucosa, making endoscopic removal difficult. Utilizing the strong magnetic attraction from the first bead, the second bead was successfully pulled out. The remaining two beads were slid into the horizontal part of the duodenum and then retrieved without difficulty ( Fig. 2 ).

**Fig. 2:**
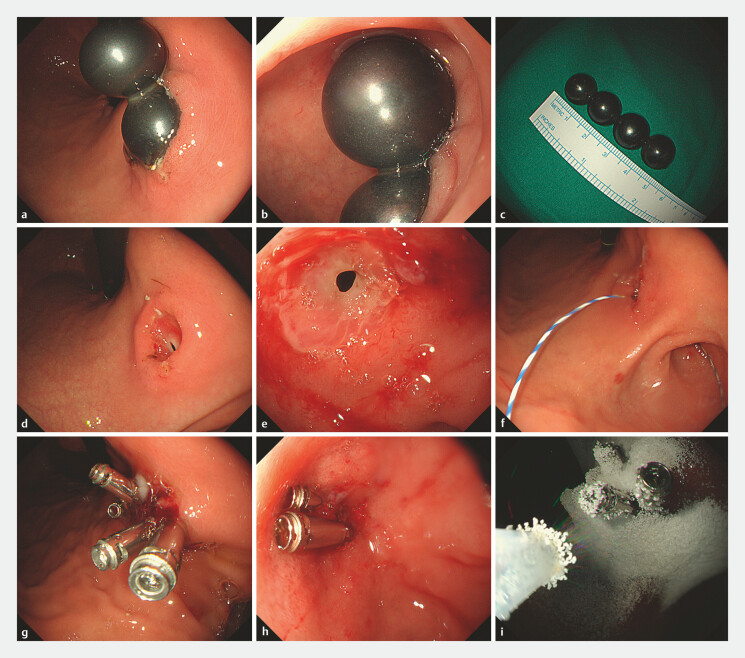
Endoscopic images. **a** Two magnetic foreign bodies were identified on the lesser curvature of the stomach, with one being deeply embedded in the gastric mucosa. **b** Two additional magnetic foreign bodies were found in the duodenal bulb. **c** The removed four magnetic foreign bodies, each approximately 1.4 cm in diameter. **d** Following the removal of the gastric foreign bodies, a fistula was detected. **e** Perforation of the posterior wall of the duodenal bulb. **f** Gastroduodenal fistula was formed, through which a Zebra guidewire could be passed from the duodenal opening into the stomach. **g** The gastric perforation was closed using titanium clips. **h** The duodenal perforation was closed using titanium clips. **i** EndoClot medication was sprayed to assist in sealing the wound surface.

After removal of all foreign objects, a gastroduodenal fistula became visible, and a guidewire was able to pass through the fistula. Given the duodenal perforation was situated on the posterior wall of the bulb, surgical repair would have been highly invasive and relatively complex. Therefore, we opted for a minimally invasive approach, using titanium clips to close the fistula and spraying EndoClot medication at the closure site 1 ( Video 1 ). One month later, the fistula had healed ( Fig. 3 , Fig. 4 ).

Unanticipated gastroduodenal fistula induced by multiple magnetic beads: successful endoscopic management without the need for surgery.Video 1

**Fig. 3:**
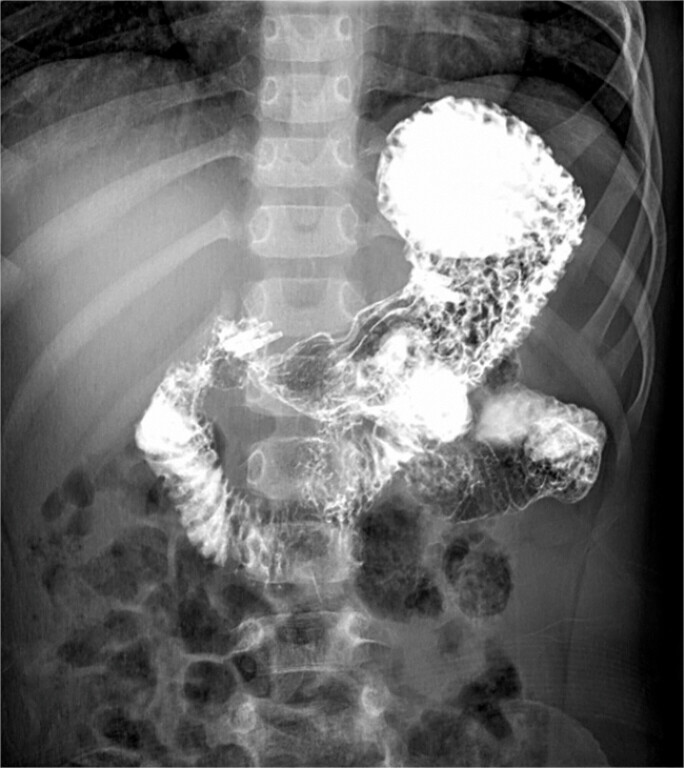
Postoperative upper gastrointestinal tract radiography at one month shows complete healing of the fistula, with no passage of contrast agent through the fistula into the duodenum.

**Fig. 4:**
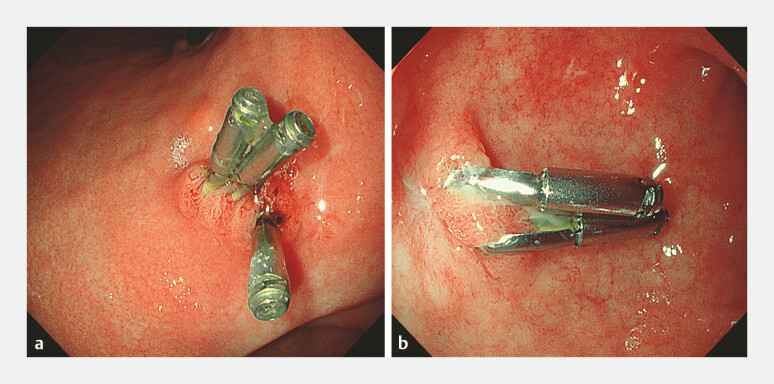
Postoperative endoscopic re-evaluation at one month demonstrates complete healing of the perforated area, with some endoclips remaining in situ. **a** Endoscopic image of the lesser curvature of the stomach. **b** Endoscopic image of the duodenal bulb.

Gastroduodenal fistula is a rare and severe complication caused by the retention of magnetic foreign bodies 2 3 . In this case, the relatively large size of the beads and their prolonged retention were considered the main contributing factors. Compared to traditional surgical repair, endoscopic intervention provides a less invasive and more effective treatment option.

Endoscopy_UCTN_Code_CPL_1AH_2AG

## Notes

Endoscopy E-Videos https://eref.thieme.de/e-videos
*E-Videos* is an open access online section of the journal *Endoscopy* , reporting on interesting cases and new techniques in gastroenterological endoscopy. All papers include a high-quality video and are published with a Creative Commons CC-BY license. Endoscopy E-Videos qualify for HINARI discounts and waivers and eligibility is automatically checked during the submission process. We grant 100% waivers to articles whose corresponding authors are based in Group A countries and 50% waivers to those who are based in Group B countries as classified by Research4Life (see: https://www.research4life.org/access/eligibility/ ). This section has its own submission website at https://mc.manuscriptcentral.com/e-videos .
